# Metabolic syndrome, diabetes and inadequate lifestyle in first-degree relatives of acute myocardial infarction survivors younger than 45 years old

**DOI:** 10.1186/s12944-017-0605-4

**Published:** 2017-11-28

**Authors:** Maria Helane C. Gurgel, Renan M. Montenegro Junior, Clarisse M. Melo Ponte, Tamara Cristina S. Sousa, Paulo Goberlanio B. Silva, Lucia de Sousa Belém, Frederico Luis Braz Furtado, Lívia A. de Araújo Batista, Alexandre C. Pereira, Raul D. Santos

**Affiliations:** 1 Heart Institute (InCor), University of Sao Paulo Medical School Hospital, São Paulo, Brazil; 2 Federal University of Ceará, Fortaleza, Brazil; 3 Christus Medical School, Fortaleza, Brazil; 4 Dr. Carlos Aberto Studart Gomes Hospital, Fortaleza, Brazil; 5 Professor Costa Mendes, 1608, Zip-code: 60416-200. Rodolfo Teófilo, Fortaleza, Ceará Brazil

**Keywords:** Myocardial infarction, Risk factors, Metabolic syndrome, Family history, Dyslipidemia, Thyroid hormones/metabolism

## Abstract

**Background:**

A premature myocardial infarction (PMI) is usually associated with a familial component. This study evaluated cardiovascular risk factors in first-degree relatives (FDR) of patients with PMI not presenting the familial hypercholesterolemia phenotype.

**Methods:**

A cross-sectional study comprising FDR of non-familial hypercholesterolemia patients who suffered a myocardial infarction <45-years age matched for age and sex with individuals without family history of cardiovascular disease. Subjects were evaluated for presence of the metabolic syndrome and its components, lifestyle, statin therapy, and laboratory parameters.

**Results:**

The sample was composed of 166 FDR of 103 PMI patients and 111 controls. The prevalence of smoking (29.5 vs. 6.3%; *p* < 0.001), prediabetes (40.4 vs. 27%; p < 0.001), diabetes (19.9 vs. 1.8%; p < 0.001), metabolic syndrome (64.7 vs. 36%; p < 0.001), and dyslipidemia (84.2 vs. 31.2%; *p* = 0.001) was greater in FDR. There was no difference on the prevalence of abdominal obesity between groups. In addition, FDR presented higher triglycerides (179.0 ± 71.0 vs. 140.0 ± 74.0 mg/dL; *p* = 0.002), LDL-cholesterol (122.0 ± 36.0 vs. 113.0 ± 35 mg/dL; *p* = 0.031), non-HDL-cholesterol (157.0 ± 53.0 vs. 141.0 ± 41.0 mg/dL; *p* = 0.004), and lower HDL-cholesterol (39.0 ± 10.0 vs. 48.0 ± 14.0 mg/dL; *p* < 0.001) than controls. Thyrotropin levels (2.4 ± 1.6 vs. 1.9 ± 1.0 mUI/L; *p* = 0.002) were higher in FDR. The risk factor pattern was like the one of index cases. Only 5.9% (*n* = 10) of FDR were in use of statins.

**Conclusions:**

FDR of non-familial hypercholesterolemia patients with PMI presented an elevated prevalence of metabolic abnormalities, inadequate lifestyle and were undertreated for dyslipidemia.

## Background

The occurrence of an acute myocardial infarction (AMI) before the age of 45 years is unusual. At the Framingham study, the 10-year incidence of AMI was 12.9:1000 in men aged 30 to 34 years and 5.2:1000 in women aged 35 to 44 years. In comparison with older individuals, the incidence of AMI is approximately 8-fold smaller in the younger population [1].

Premature coronary artery disease (CAD) is strongly associated with a familial component. Many studies have shown high proportions of cardiovascular risk factors as well as subclinical coronary atherosclerosis in first-degree relatives (FDR) of individuals with a premature myocardial infarction (PMI) [1, 2]. The importance of a positive family history as an independent biomarker for premature CAD is now clearly established [3]. Family history of CAD is usually defined as a coronary event occurring in a FDR, before ages 55 and 65 years in male and females, respectively [4]. However, there is little information regarding lifestyle factors that predispose to cardiovascular disease in FDR of AMI survivors younger than 45 years of age.

Familial hypercholesterolemia has been associated with PMI and there has been a renewed interest on this disease due to the development of newer treatments to reduce LDL-cholesterol (LDL-C) [5], however its presence explains only a minority of PMI cases [6]. Therefore, the aim of this study was to evaluate CAD risk factors in FDR of Brazilian patients who suffered a myocardial infarction before age 45 years who did not present the familial hypercholesterolemia phenotype.

## Methods

### Study design and subjects

This is a cross-sectional, single-center study. We consecutively selected index cases aged <45 years, from both sexes, diagnosed with an AMI according to the American Heart Association criteria [7]. All included patients presented CAD confirmed by angiography, and were screened between September 2011 and January 2015, at the cardiometabolism outpatient unit of the Hospital do Coração Dr. Carlos Alberto Studart Gomes (Messejana hospital), a tertiary cardiology hospital in Fortaleza, Brazil. Subjects with diagnosis of thyroid dysfunction; with suspicion of familial hypercholesterolemia defined as LDL-C > 190 mg/dL or the presence of cutaneous or tendinous xanthomas; using corticosteroids, immunosupressants, or illicit drugs; as well as pregnant or lactating women were excluded.

We invited the FDR of all index subjects to participate in the study through letter or telephone contact to perform a clinical and laboratory evaluation. Asymptomatic sex and age volunteers without family history or premature CAD were used as controls.

### Clinical evaluation

All index patients, FDR and asymptomatic volunteers were interviewed and examined by the same physician (MHG). The following risk factors were assessed: (1) smoking: current smokers and ex-smokers who had quit smoking for less than 3 years were considered smokers [8]; (2) dyslipidemia: classified as isolated hypercholesterolemia (LDL-cholesterol − LDL-C ≥ 160 mg/dL), isolated hypertriglyceridemia (triglycerides – TG ≥150 mg/dL), mixed hyperlipidemia (LDL-C ≥ 160 mg/dL and TG ≥150 mg/dL), and low HDL-cholesterol (HDL-C) values (isolated, males <40 mg/dL and females <50 mg/dL; or associated with high levels of LDL-C or TG) [9]; (3) hypertension: patients using antihypertensive medication or with a history of systolic blood pressure > 140 mmHg and/or diastolic blood pressure > 90 mmHg previously assessed at least during 3 different occasions [10]; (4) abnormal glycemic status: presence of diabetes mellitus (glycated hemoglobin − HbA1c ≥6.5%, or fasting plasma glucose ≥126 mg/dL, or 2-h plasma glucose ≥200 mg/dL during an oral glucose tolerance test, or a random plasma glucose ≥200 mg/dL in patients with classical symptoms of hyperglycemia or hyperglycemic crisis), or individuals at high risk for developing diabetes (impaired fasting plasma glucose value between 100 and 125 mg/dL), or impaired glucose tolerance: 2-h plasma glucose in 75-g oral glucose tolerance test value of 140 to 199 mg/dL, or HbA1c 5.7 to 6.4%), according to the American Diabetes Association [11]; (5) excess body weight: overweight (body mass index − BMI ≥25.0 to 29.9 kg/m^2^) and obesity (BMI ≥30.0 kg/m^2^) [12]; (6) sedentarism: physical activity of less than 150 min per week [13]; (7) metabolic syndrome: presence of at least three criteria defined by the International Diabetes Federation; abdominal obesity defined as waist circumference > 90 cm in males, >80 cm in females or BMI ≥25 kg/m^2^; fasting TG levels ≥150 mg/dL; HDL-C < 40 mg/dL in males and <50 mg/dL in females; elevated blood pressure defined as values ≥130/85 mmHg or current use of antihypertensive drugs; impaired fasting glucose defined as fasting plasma glucose ≥110 mg/dL or use of antidiabetic medications or previous history of type 2 diabetes [14].

### Anthropometric assessment

Anthropometric assessment consisted of fasting weight determination after urination, with use of light clothing and no shoes, measured with calibrated scales; height was measured without shoes in a calibrated stadiometer; BMI was derived from weight and height; abdominal circumference was measured at the midpoint between the last rib and the anterior iliac spine, with a metric tape in parallel to the ground.

### Hard coronary heart disease risk estimation

The 10-year hard coronary heart disease risk estimation was calculated using Framingham risk score equations [15] in FDR and controls. Subject were stratified as high risk (>20%); intermediate risk (10 to 20%); and low risk (<10%).

### Laboratory evaluation

The following laboratory blood tests were performed in study subjects: fasting blood glucose, total plasma cholesterol, HDL-C, LDL-C, TG and thyroid stimulant hormone (TSH). Fasting plasma glucose was evaluated using the enzymatic colorimetric hexokinase method. TG and total plasma cholesterol were measured using an enzymatic colorimetric method with cholesterol esterase oxidase and glycerol phosphate oxidase, respectively. HDL-C was assayed using the enzymatic colorimetric method with polyethylene glycol. LDL-C was calculated using the Friedewald formula for TG levels <400 mg/dL. Non-HDL-C was calculated by the formula: Total cholesterol - HDL-C. All analyses were performed in the hospital’s central laboratory using Roche Diagnostic commercial kits by the multichannel automatic analyzer Roche Cobas™ 6000.

### Statistical analysis

Continuous variables are expressed as mean ± standard deviation (SD) and normality was tested, using the Kolmogorov-Smirnov test, followed by analysis with Student’s t test (parametric data) or Mann-Whitney’s test (nonparametric data). Categorical data are expressed as absolute frequency and percentages, and were tested by the chi-square test for bivariate analysis. Results were considered significant when *p* < 0.05.

## Results

**Fig. 1:**
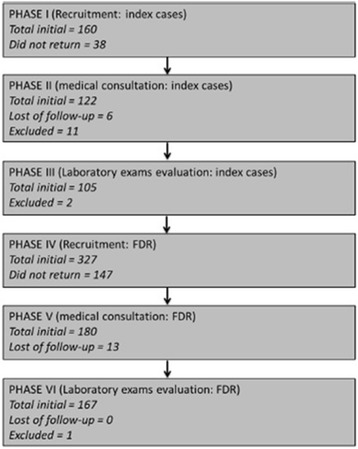
Study subject inclusion and exclusion algorithm

Figure 1 shows patient recruitment algorithm. A total of 103 index cases and 327 FDR were invited. The reasons for non-inclusion were: no response to invitation (*n* = 147) and lack of laboratory sample collection (n = 14). Hypothyroidism was an exclusion criteria for one participant. A total of 166 asymptomatic volunteers were enrolled (aged between 18 to 70 years) in the FDR group. The mean age of FDR and controls were 43.6 ± 12.6. and 41.5 ± 6.5 years (*p* = 0.148) respectively. There was a predominance of female sex in both groups 63.2% (*n* = 105) and 68.4 (*n* = 76) respectively in FDR and control groups (*p* = 0.278).

Table 1 shows clinical and laboratory characteristics of index cases. There was an elevated prevalence of obesity, sedentarism, smoking, diabetes and hypertension. More than 80% of index cases fulfilled the criteria for the metabolic syndrome. The most frequent dyslipidemia patterns were low HDL-C levels and hypertriglyceridemia despite the use of statins by 90% of patients. Of importance 42% of index cases presented a history of early CAD in the family and only 18.6% (*n* = 19) had LDL-C < 70 mg/dL. Table 2 shows the comparison of clinical and laboratory characteristics between FDR and controls according to sex. The prevalence of dyslipidemia, mainly low HDL-C levels and hypertriglyceridemia, smoking, pre-diabetes, diabetes, and the metabolic syndrome was higher in FDR individuals than in controls (all parameters *p* < 0.001). However, there were no differences concerning the prevalence of hypertension, abdominal obesity and overweight. Only 5.9% (*n* = 10) of FDR and none of the controls were in use of statins.

**Table 1:** Clinical and laboratory characteristics of 103 patients <45 years with myocardial infarction

Parameters	(*n* = 103)
Age (years)	39.6 ± 5.7
Male sex n (%)	56.0 (54.4)
Overweight n (%)	86.0 (86.0)
Abdominal obesity n (%)	87.0 (88.8)
Dyslipidemia n (%)	96.0 (93.2)
Hypertension n (%)	44.0 (42.7)
Sedentarism n (%)	87.0 (84.3)
Smoking n (%)	59 (57.3)
High LDL-C (mg/dL)	5.0 (5.1)
Low HDL-C (mg/dL)	87.0 (84.5)
High triglycerides (mg/dL)	63.0 (61.2)
Metabolic syndrome n (%)	83.0 (82.2)

**Table 2:** Clinical and laboratory characteristics of first-degree relatives (FDR) of patients with premature acute myocardial infarction and individuals with no family history of coronary artery disease (controls)

Parameters	FDR		Controls		p-value^a^ FDR vs control
	(*n* = 166)		(*n* = 111)		
	Female (*n* = 105)	Male (*n* = 61)	Female (*n* = 63)	Male (*n* = 48)	
Overweight n (%)	78 (78)	46 (80.7)	43 (56.5)	25 (71.4)	0.051
Abdominal obesity n (%)	92 (93.8)	40 (71.4)	66 (86.8)	28 (80)	0.976
Smoking n (%)	29 (29.6)	20 (32.7)	1 (1.3)	5 (14.2)	<0.001
Sedentarism	105 (63.2)	61 (36.7)	63 (56.8)	48 (43.2)	1.000^b^
Hypertension n (%)	27 (25.7)	15 (24.5)	14.4 (11)	40 (12)	0.878
Diabetes n (%)	15 (53.7)	13 (46.4)	1.3(1)	3 (1)	<0.001
Prediabetes n (%)	61.6 (40.4)	23 (38.3)	16 (21.0)	10 (28.5)	0.024
Dyslipidemia n (%)	88 (62.8)	52 (37.1)	38 (50)	29 (82.8)	<0.001
LDL-C > 160 mg/dL n (%)	17 (16.5)	6 (10.9)	5 (6.5)	3 (8.5)	0.045
Low HDL-C n (%)	87 (82.8)	42 (68.8)	35 (46)	26 (74.2)	<0.001
TG >150 mg/dL n (%)	42 (40)	40 (65.5)	16 (21)	17 (48.5)	0.003
Metabolic syndrome n (%)	42 (40)	29 (47.5)	15 (19.7)	14 (40)	<0.001

^a^Chi-square test (FDR versus controls considering the whole population (independent of sex)^b^Fisher exact test

Table 3 shows the comparison of laboratory parameters between FDR and controls. TG (*p* = 0.002), LDL-C (*p* = 0.031), non-HDL-C (*p* = 0.004), and TSH levels (p = 0.002) were higher, and HDL-C concentrations were lower (*p* < 0.001) in FDR than in controls.

**Table 3:** Comparison of laboratory parameters between first-degree relatives (FDR) of premature myocardial infarction individuals and controls

Variables	FDR (*n* = 166)	Controls (*n* = 111)	*p*-value^a^
Fasting blood glucose (mg/dL)	103 ± 33	94 ± 13	0.521
Total cholesterol (mg/dL)	196 ± 44	190 ± 39	0.219
Non-HDL-C (mg/dL)	157 ± 43	141 ± 41	0.004
LDL-C (mg/dL)	122 ± 37	113 ± 35	0.031
HDL-C (mg/dL)	39 ± 10	48 ± 14	<0.001
Triglycerides (mg/dL)	179 ± 71	140 ± 74	0.002
TSH (mUI/L)	2.4 ± 1.6	1.9 ± 1	0.002

^a^Mann-Whitney test

**Fig. 2:**
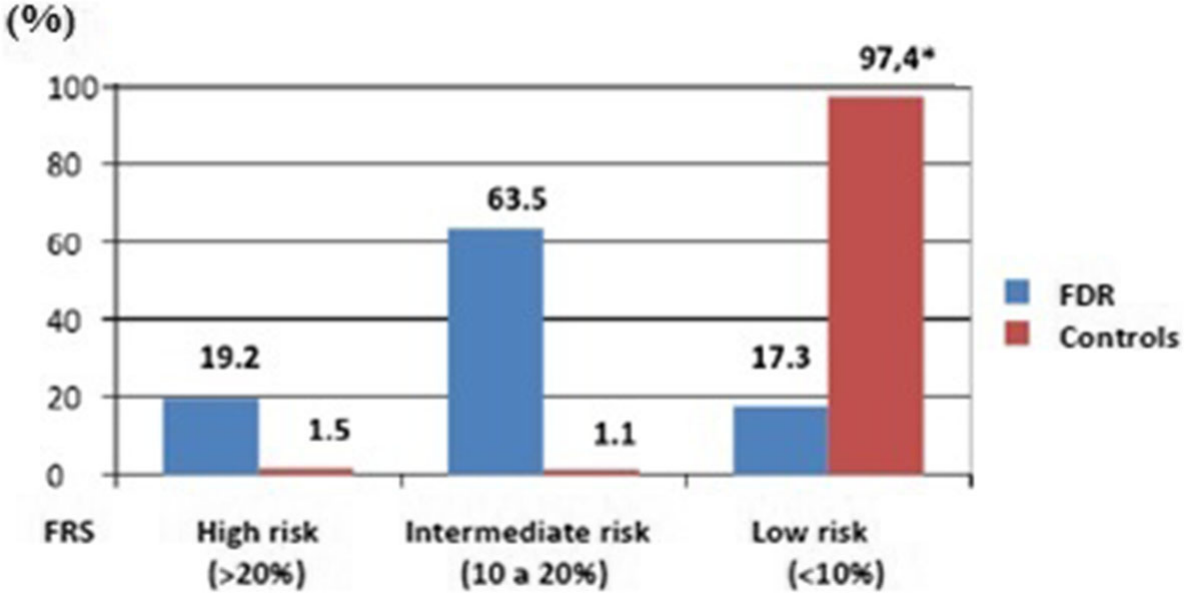
Predicted 10-year Framingham hard coronary heart disease risk of first degree relatives of index cases (*n* = 166) and controls (*n* = 111). Results are expressed in %. Test used: chi-square. * *p* < 0.001. FRS: Framingham risk score; FDR: first degree relatives

Figure 2 shows the comparison of estimated 10-year hard coronary heart disease risk between FDR and controls. Roughly 83% of FDR were classified as high/intermediate risk vs. 3% of controls (p < 0.001).

## Discussion

In this study, FDR of subjects, without clinical suspicion of familial hypercholesterolemia, who suffered an AMI before the age of 45 years presented an elevated burden of cardiovascular risk factors mainly type 2 diabetes, pre-diabetes, atherogenic dyslipidemia, metabolic syndrome, smoking and sedentarism in comparison with controls. Of importance FDR individuals presented a very similar risk profile to the one of index cases, a fact indicating that genetic and or lifestyle factors are shared within the families. In addition, despite the elevated prevalence of dyslipidemia and a great number of individuals considered as intermediate/high hard coronary heart disease risk, almost 95% of them were not in use of preventive therapies like statin treatment.

Studies evaluating the prevalence of atherosclerosis risk factors among patients with premature CAD have demonstrated a high prevalence of lipid abnormalities, abdominal obesity, hypertension, diabetes and smoking, when compared to healthy controls. In the present study, a similar pattern was found. This occurred despite a similar prevalence of excess body weight and abdominal obesity between FDR and controls, a pathologic condition associated with most previously cited risk factors except smoking.

The present study also encountered higher levels of TSH (even within the normal reference value) in FDR individuals compared with controls, a finding not previous described. Previous studies evaluated the association of TSH (in the upper limit, but within a normal reference value) with components of the metabolic syndrome [16]. The pathophysiological mechanism of this association is still unclear, but it is known that the myocardium and vascular endothelial tissue have receptors for thyroid hormones and are sensitive to changes in their serum concentrations. Even minor variations in such concentrations could lead to a negative impact on the cardiovascular system [17, 18].

The National Health and Nutrition Examination Survey III (NHANES III) suggested the reference value for TSH in the general population is between 0.4 and 4.12 mU/L [19]. However, there is evidence that TSH levels between 2.5 and 4.0 mU/L are related to metabolic alterations, and the discussion about normal TSH upper limit is increasing. Also, the National Academy of Clinical Biochemistry ratifies that over 95% of healthy euthyroid individuals present TSH concentrations between 0.4 and 2.5 mU/L. Thus, these considerations have raised the discussion that the upper limit of normal TSH values should be reduced to 2.5 mU/L [20].

Several studies reported elevated levels of cholesterol in individuals with subclinical hypothyroidism that were reverted with the replacement of levothyroxine [21, 22]. The HUNT Study (Nord-Trøndelag Health Study), which evaluated the association between TSH levels within the normal reference interval and serum lipid concentrations, showed a positive and linear association of total cholesterol, LDL-C, non-HDL-C and TG with TSH, and a negative relation with HDL-C levels [23]. Whether the encountered elevated TSH levels contributed to the high atherosclerosis risk factor burden encountered in FDR of PMI patients in this study needs further evaluation.

One important finding of our study was that despite statins were used by 90% of index cases, LDL-C was not adequately controlled and there was a marked presence of residual atherogenic dyslipidemia with predominance of low HDL-C concentrations. This pattern was similar in FDR where 85% of individuals presented dyslipidemia and 73% had low HDL-C. These findings corroborate previous studies that also reported this phenotype in individuals with early coronary heart disease onset and in their families [3, 24]. Indeed, this pattern has been previously described to the so called Familial Combined Hyperlipidemia (FCH) phenotype [25]. FCH has been used as a term to describe a group of individuals within the same family with a phenotype of mixed dyslipidemia, or isolated hypercholesterolemia, presenting or not low HDL-C and with an early onset of cardiovascular disease. The dyslipidemia pattern is variable among members of the same family and is influenced by diet, exercise and weight status. In addition, many of the so called “FCH patients” also developed dysglycemia, type 2 diabetes and hypertension. However, the lack of a common and homogeneous genetic background in affected families has casted doubt on the very existence of that condition [26]. Indeed, a consensus document of the European Atherosclerosis Society on hypertriglyceridemic states did not consider the existence of FCH as a cause of hypertriglyceridemia or mixed dyslipidemia [27]. However, this does not exclude a possible genetic component for the common phenotype of index cases and FDR.

This study has several limitations: its cross-sectional design; difficulties for FDR’s adherence to attend medical appointments and exams, and the consequent loss of 55% of relative patients; the evaluation of thyroid function in FDR did not include the analysis of free T4 and anti-thyroid antibody profile. However, its value is related to study of individuals with AMI at a very early age and clearly shows a similar atherosclerosis risk factor pattern in index cases and FDR that was clearly more severe than in controls.

## Conclusions

FDR of patients with PMI not presenting the familial hypercholesterolemia phenotype had an unfavorable metabolic profile characterized by the elevated presence of type 2 diabetes, atherogenic dyslipidemia, metabolic syndrome and its components. The metabolic abnormalities were like the ones of index cases and the predicted cardiovascular risk was significantly higher than in controls. In addition, these individuals were severely undertreated regarding dyslipidemia control. This study clearly shows clustering of risk factors in relatives of individuals with PMI. FDR presenting those abnormalities must be submitted to intensive cardiovascular disease risk factor modification programs.

## Abbreviations

AMI: Acute myocardial infarction
BMI: Body mass index
CAD: Coronary artery disease
FCH: Familial combined hyperlipidemia
FDR: First-degree relatives
HbA1c: Glycated hemoglobin
HDL-C: High-density lipoprotein-cholesterol
LDL-C: Low-density lipoprotein-cholesterol
PMI: Premature myocardial infarction
SD: Standard deviation
TG: Triglycerides
TSH: Thyroid stimulant hormone
